# Shifts and importance of viable bacteria in treatment of DSS-induced ulcerative colitis mice with FMT

**DOI:** 10.3389/fcimb.2023.1124256

**Published:** 2023-02-06

**Authors:** Jinglong Liu, Hao Lin, Man Cao, Tan Lin, Aiqiang Lin, Wei Xu, Han Wang, Jianquan He, Yuantao Li, Hailing Tang, Bangzhou Zhang

**Affiliations:** ^1^Department of Gastroenterology, Shanxi Provincial People’s Hospital, Taiyuan, China; ^2^Center for Microecological Medical Technology, Xiamen Institute of Union Respiratory Health, Xiamen, China; ^3^Center for Research and Development, Xiamen Treatgut Biotechnology Co., Ltd., Xiamen, China; ^4^Department of Gastroenterology, The Second Affiliated Hospital of Fujian University of Traditional Chinese Medicine, Fuzhou, China; ^5^Division of Gastroenterology, Xi’an Central Hospital, Xi’an, China; ^6^School of Pharmacy, Fujian University of Traditional Chinese Medicine, Fuzhou, China

**Keywords:** viable gut microbiota, PMA, FMT, DSS-induced colitis, 16S rRNA gene sequencing

## Abstract

**Background and Aims:**

Ulcerative colitis (UC) has become a global public health concern, and is in urgent need of novel therapies. Fecal microbiota transplantation (FMT) targeting gut microbiota has recently been applied to the treatment of UC. Despite its recent successes, it is still largely unknown how FMT functionally modulates the gut microbiota and improves the disease.

**Methods:**

We prospectively collected fecal samples from the 40 mice (30 mice for dextran sulfate sodium (DSS)-induced, 10 for controls), followed by Propidium monoazide treatment for 16S rRNA gene sequencing. These 30 mice were divided equally into 3 groups, which were transplanted with original donor microbiota (DO), inactivated donor microbiota (DI) and saline, respectively. Subsequently, we used 16S rRNA gene sequencing to analyze the viable gut bacteria of ulcerative colitis (UC) mice and histological analysis to evaluate the effects of fecal microbiota transplantation (FMT) with viable microbiota.

**Results:**

We demonstrated that the community structure of viable bacteria was significantly different from fecal bacteria based on total DNA. Furthermore, the intestinal viable microbiota and colonic mucosal structure of mice were significantly changed by DSS induction. The histological analysis showed that only the mice treated with original donor microbiota group (HF) achieved a significant improvement. Compared with inactivated donor microbiota group (IF) and saline (NF), Lactobacillus and Halomonas were significantly enriched in the HF group.

**Conclusion:**

We inferred that only live bacteria from human donor reversed the histopathology and symptoms of UC in mice and altered the gut microbiota. The activity of gut microbiota in donor samples should be considered in FMT and that detailed analysis of viable microbiota is essential to understand the mechanisms by which FMT produces therapeutic effects in the future.

## Introduction

Ulcerative colitis (UC) is one subtype of inflammatory bowel disease (IBD) that has a high incidence and prevalence in the worldwide (Lima et al., 2021). It is also thought to be intricately caused by variable factors, including genetic (Hedin et al., 2015), immunological and environmental aspects (Braun and Wei, 2007), of which the gut microbiota dysbiosis may play important roles (Sheehan et al., 2015; Kump et al., 2018; Zhang et al., 2022). Despite available therapies, including corticosteroids, anti-tumor necrosis factor alpha (TNF-α) agents, aminosalicylates, immunomodulators, and surgery (Weisshof et al., 2018), development of new therapies and investigation of alternative strategies are in urgent need for amount of patients who are unresponsive to these existing treatments or present secondary failure during treatment.

Fecal microbiota transplantation (FMT) is a novel treatment method which is to transfer the functional microbiota from normal feces to an unbalanced gastrointestinal tract, reconstruct a new intestinal flora, and resume the host function (Costello et al., 2017; Liu et al., 2021) This technique has proven effective impact in many microbiota-related metabolic (Lee et al., 2019; Que et al., 2021; Gong et al., 2022), infectious (Smillie et al., 2018), and inflammatory diseases (Weingarden and Vaughn, 2017; Zhang et al., 2019). In recent research, the patients have recurrent *Clostridium difficile* infection were extremely effective treated by FMT (about 90% cure rate) (Le Bastard et al., 2018). However, the effectiveness of FMT varied among different studies (Li et al., 2016; Smillie et al., 2018), in which specified donors may play crucial roles. These differences not only exist between individuals but sometimes even within a same person (McOrist et al., 2011). For example, one study showed no improvement in clinical and endoscopic remission at 12 weeks following two infusions of FMT from healthy donors *via* a nasogastric tube, while another study showed higher endoscopic remission at 7 weeks in patients treated with weekly FMT enemas (Moayyedi et al., 2015; Rossen et al., 2015). Therefore, the detailed analysis of the composition and numbers of microbiota transplanted is essential to understand the differences in therapeutic effectiveness and mechanisms of FMT from donor samples.

The gut microbiota is a complex ecosystem with a range of bacterial genera that perform many important functions in the host, including maintaining gut homeostasis, intestinal epithelial barrier, immune system development and providing essential metabolic substrates for colon cells (Pushalkar et al., 2018), play important roles in UC progress (Ni et al., 2017; Liu et al., 2021). Gut microbiota manipulation by FMT has demonstrated promising effectiveness in UC remission in experimental colitis mice model trials. For instance, Zhang et al. evaluated the FMT effect on the composition of the colonic microbiota to determine whether changes in the gut microbiota were associated with the protective effect of FMT in DSS-induced mice (Zhang et al., 2022). Lima et al. identified the speices *Odoribacter splanchnicus*, which plays a key role in FMT, by performing the immune response use omic analysis in donor and recipient fecal samples (pre- and post-intervention), Further through mouse experiments, they proved that *Odoribacter splanchnicus* is the key bacterium (Lima et al., 2021). Similarly, Li et al. treated a mouse model of DSS-induced colitis with FMT in combination with a 16S rRNA analysis, revealing that FMT ultimately alleviates colitis by regulating the flora (Li et al., 2021a). Generally, in all of these analyses, the changes in metagenome-based strategy include 16S rRNA sequencing of total bacteria DNA were observed, while changes in the live bacteria DNA were neglected. Moreover, with the deepening of the study, living microbiota are considered to be therapeutic agents for FMT, because the colonization of these microbiota in the intestines of recipients may lead to lasting changes in patients (Khoruts et al., 2010; Seekatz et al., 2014). Therefore, it is of great significance to focus on the viable bacteria for understanding the mechanism of FMT and exploring the crucial gut microbiota.

In this paper, we presented a pioneer work to evaluate the living bacteria of gut microbiota from UC patients by FMT trials. First, 40 mice were collected for analysis. Then, 30 of them were induced with colitis by DSS and 10 were not treated with DSS served as controls. These 30 mice were also divided equally into 3 groups. Group one was transplanted with initial donor microbiota (HF), while group two was transplanted with inactivated flora (IF), and group three were transplanted with saline (NF). Subsequently, the remission rate of FMT treatment was analysed by histopathology and symptoms in mice. Meanwhile, PMA-treated donor samples were analysed by 16S rRNA gene sequencing for structural changes of total and viable bacteria DNA, as well as changes in live bacteria between DSS and controls, pre- and post- FMT, respectively. This method can accurately evaluate the changes in viable bacteria in the gut microbiota, establish a methodological basis donor screening, evaluation before and after transplantation of viable bacteria in fecal samples in the future.

## Materials and methods

### Preparation of donor stool sample

Samples were collected with informed consent from all participants. All participants completed a questionnaire-based interview and underwent a physical examination for screening of donors (He et al., 2021). Every subject provided fresh stool samples in a stool container on site. Fecal microbiota were extracted with an automatic fecal microbiota extractor TG-01 Extn (Treatgut, Guangzhou, China) in Xiamen Treatgut Biotechnology Co. Ltd. Fecal sludge (FS) were collected by centrifugation at 5,000 g for 5 min. The collected microbiota were then added to saline at a ratio of 1:1.1, and half of the resulting solution were autoclaved in a 250 mL Erlenmeyer flask at 121 °C for 30 min to prepare inactivated donor microbiota (DI), with the remainder serving as the original donor microbiota (DO). Total microbiota andviability were determined by flow cytometry with LIVE/DEAD™ BacLight™ Bacterial Viability Kit (Thermo Fisher Science). Analyses were carried out using a BD Accuri™ C6 Plus Flow Cytometer (BD Biosciences, USA) system. Meanwhile, the PMA-qPCR standard curve of donors were established.

### Animals and experimental design

A total of 40 male C57BL/6J mice (7 weeks old, 18-20 g weight) were purchased from the Gempharmatech Co., Ltd (China). Mice were allowed one week to acclimate prior to the study. For this period, food and water were given ad libitum and the room was ventilated, having an ambient temperature of 22 °C ± 1°C with 50% ± 10% humidity and a 12-h diurnal light cycle (lights on 07:00–19:00). 30 of 40 mice were administered a 3% dextran sulfate sodium (DSS, MP Biomedicals, USA) solution and 10 for controls not treated with DSS. These 30 DSS-induced mice were divided equally into 3 groups, which were HF, IF and NF, respectively. Mice were treated with DSS for 5 days and then gavaged for 3 days. 200 μL per dose once daily for 3 days in the HF and IF groups, and equal saline doses in the NF and control groups. Fresh fecal (250 mg) from mice in four groups were collected at the 7, 12 and 15 days, and resuspended in a 5 ml saline, vigorously shaken 3 min for subsequent analyses. All animal experiments reported in this study were approved by the Animal Care and Ethics Committee of Fujian University of Traditional Chinese Medicine Laboratory Animal Center.

### Phenotype detection of mice

During the intervention period, the body weight and stool consistency of mice were observed regularly. DAI scoring criteria refers to Rangan et al. (Rangan et al., 2019). After intervention, animals were humanely sacrificed by cervical dislocation, and the colons were removed. Colon lengths and weights were measured using a ruler and an electronic analytical balance respectively. To observe detailed histopathological changes, the colons of different mice were first stored in a 10%buffered formalin solution. These were then embedded in paraffin, cut into 5 μm sections, stained with hematoxylin-eosin, and then placed under a light microscope for examination.

### PMA-treated samples

Stock solution was prepared by dissolving 1 mg of PMA (US Everbright Inc,Suzhou,China) in 1 mL of 20% dimethyl sulfoxide. For PMA treatment, the FS samples treated as above method was diluted 100 times in normal saline solution. A 487.5 μL solution was weighed and transferred to an aseptic EP tube, followed by the addition of 12.5 μL PMA solution. The solution was mixed, and the tubes were incubated in dark for 10 min at room temperature. Samples were exposed to an LED light (500W) with periodic mixing at a distance of 15 cm for 10 min. In non-PMA treated control aliquots, 12.5 μL saline was added instead of PMA. Control samples underwent identical incubation and light-exposure as the matching PMA treated samples (Emerson et al., 2017). All samples was treated by PMA for further analysis.

### DNA extraction

DNA was extracted from fecal samples using the QIAamp Fast DNA Stool Mini Kit (Qiagen, CA, USA) flowing the manufacturer’s instructions. The concentration and purity of the isolated DNA was assessed using spectrophotometry (Multiskan™ GO, Thermo Fisher Scientific, USA). The DNA extracts were also evaluated for quality by agarose (1.5%) gel electrophoresis in 1× Tris-Acetate-EDTA buffer. DNA samples were stored at -20˚C before being used as templates for next-generation sequencing library preparation.

### Library preparation and sequencing

Sequencing libraries were generated using TruSeq^®^ DNA PCR-Free Sample Preparation Kit (Illumina, USA) following manufacturer’s recommendations and index codes were added. The library quality was assessed on the Qubit@ 2.0 Fluorometer (Thermo Fisher Scientific, Waltham, MA, China) and Agilent Bioanalyzer 2100 system. At last, the library was sequenced on an Illumina MiniSeq 150 bp paired-end reads were generated.

### Quantitative PCR

Bacterial 16S rRNA genes in the fecal samples were quantified using real-time qPCR on a StepOnePlus Real-Time PCR system (Thermo Fisher Scientific, Waltham, MA, China). The V4 variable regions of bacterial 16S rRNA gene were PCR-amplified using the primers (515F 5’-GTGYCAGCMGCCGCGGTAA-3’,806R 5’-GGACTACNVGGGTWTCTAAT-3’). Each reaction mixture had a total volume of 20 µL. Itcontains 2 µL of sample DNA, 10 µL of ChamQ Universal SYBR qPCR Master Mix (Vazyme Biotech, NJ, China), 0.4 µL of each 10µM primer, and 7.2 µL of sterilized ultra-pure water. The cycle conditions of the real-time PCR were as follows: initial holding at 95 °C for 30 s, 40 cycles of denaturation at 95 °C for 10 s followed by annealing/elongation at 60 °C for 30 s. The specificity was determined after amplification by a melting curve analysis. All qPCR tests were performed in triplicate, and the mean values were used for analysis.

### Bioinformatics and statistical analysis

First, Fast Length Adjustment of Short Reads (FLASH) (V1.2.11) was used to assemble paired-end reads for the V4 region, the -x 0.15 option was selected to control the maximum mismatched base pairs ratio in the overlap area, and the -M 150 option was selected to control the maximum length of the overlap area. Then, cutadapt (V1.13) was used to trim and filter the sequence data processed from FLASH, including removing adapter sequences and discarding sequences with fewer than the specified number of bases. Subsequently, sequences were quality filtered by Usearch with the -fastq_maxee 1.0 option. After quality control, unique sequences were obtained by eliminating redundancy, and they were sorted in descending order according to sequence abundance. Meanwhile, singletons in the sequence data were removed. To assign denovo OTUs, we removed chimeric sequences and clustered sequences with 97% similarity and using Usearch (Edgar, 2013) for individual study. The representative sequences of OTUs were aligned to the SILVA 132 database for taxonomic classification by RDP Classifier (Wang, 2007) and aggregate to various taxonomic levels.

Based on the OTU tables derived from each sample, alpha-diversity indices between every sample were calculated, including bacterial richness (observed OTUs), shannon index, and evenness (J). Significance tests of alpha-diversity indices were conducted by the Wilcoxon test method. Then, Principal coordinates analysis (PCoA) based on Bray-Curtis distance at the OTU level was utilized for beta-diversity to visualize the differences in microbial community structure across samples. Significance tests of beta-diversity indices were determined using permutational multivariate analysis of variance (PERMANOVA) with 10^4^ permutations in vegan. Linear discriminant analysis (LDA) effect size (LEfSe) was employed to identify the taxa most likely to explain the differences between groups. LEfSe uses a nonparametric Kruskal–Wallis rank sum test to assess different features with significantly different abundance between assigned taxa and performs LDA to estimate the effect size of each sequence variant, as reported by (Segata et al., 2011). Finally, the results were visualizing using the custom R script based on ggplot2 (Wickham et al., 2016). These analyses were performed using R v3.4.1, GraphPad Prism and SPSS software. A *p* value < 0.05 was considered statistically significant. In addition, all obtained data are expressed as the mean ± standard deviation (SD).

## Results

### Therapeutic effect of live microbiota in mice with DSS colitis by FMT

**Figure 1:**
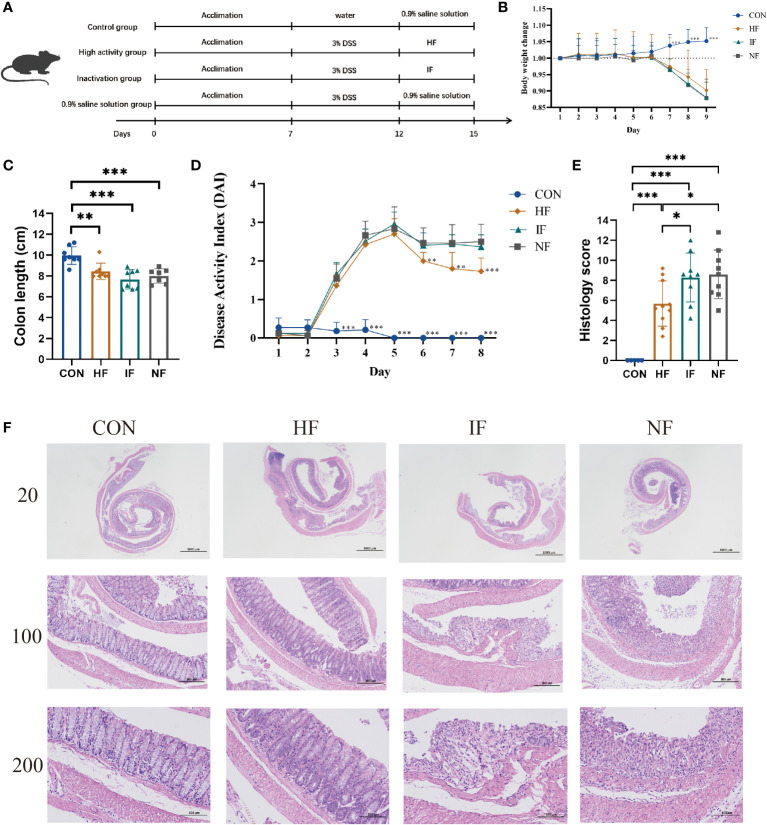
**(A)** The animal experimental protocol. **(B)** Daily body weight changes throughout the entire duration of the study. **(C)** the lengths of colon from each group. **(D)** Kinetics of DAI scores throughout the entire duration of the study. **(E)** Histological scores of colons. **(F)** H&E stained colon sections. Data are presented as mean ± SD. ***p < 0.001, **p < 0.01 and *p < 0.05 vs the NF group.

To investigate the alleviating effect of live FMT bacteria on colitis, we induced experimental colitis in mice (n=30) by administering 3% DSS in water for 5 consecutive days and then started FMT intervention in mice on the sixth day for 3 consecutive days (**Figure 1A**). The control (CON) group of mice (n=10) were in good mental condition, without diarrhoea and soft stools. The DSS mice had loose stools from day 3 of the moulding, followed by severe soft stools, bloody stools and depression on day 4. The DSS-induced mice were divided equally into three groups for FMT of DO, DI feces and saline treatment. From the **Figure 1B**, we could see that there was no significant difference among the HF, IF and NF groups, all of which showed a decreasing trend in body weight. Colonic length were significantly decreased compared to CON group, while there was no significant difference among the HF, IF and NF groups (**Figure 1C**). The DAI scores of DSS-induced mice increased on day 3, with the mice in the HF group had significantly lower DAI scores than NF group (**Figure 1D**). In terms of histological scores, the HF group was significantly lower than both the IF and NF groups, while there was no significant difference between the IF and NF groups (**Figure 1E**). Meanwhile, the histological analysis further revealed that the histopathological status of the observed colonic specimens was as shown in the **Figure 1F**, with normal colonic tissue morphology in the CON and HF groups, with a clear hierarchy of tissue structures, with the mucosal, submucosal and muscular layers clearly visible and the crypt and cupped cells well arranged. A variable number of inflammatory cells were seen, and the crypt was dilated near the ulcer foci. In summary, these results demonstrate that live bacteria in FMT are able to participate in and improve clinical colonic inflammatory conditions and colonic damage, whereas dead bacteria do not.

### Differences between bacterial communities from total fecal DNA and PMA-treated DNA

**Figure 2:**
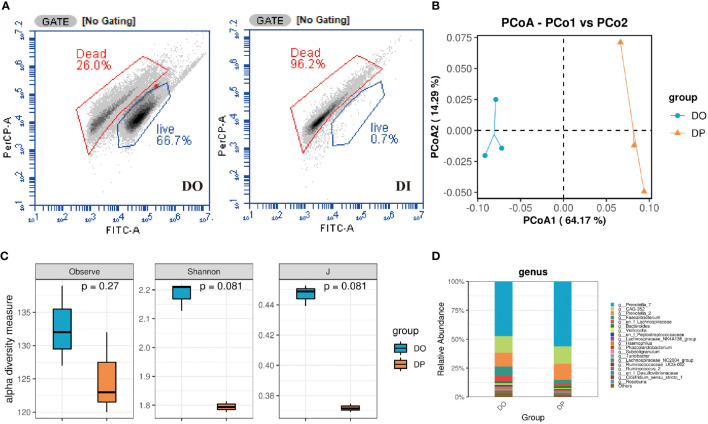
Composition of donor gut microbiota before and after PMA treatment turned out. **(A)** Flow cytometry counts of DO and DI groups. DO, DI and DP samples were analyzed based on 16S rRNA gene sequencing. **(B)** PCoA analysis; **(C)** Taxonomic profiles at the top 20 genus levels in terms of overall mean relative abundance; **(D)** microbial alpha diversity as estimated by species richness, the Shannon diversity index, and the Inverse Simpson diversity index(J) based on OTU abundance data.

Flow cytometric analysis showed that the original donor microbiota retained roughly 66.7% of viable bacteria, while almost all of the viable bacteria were removed after the heat-killed treatment, which is less than 0.7% (**Figure 2A**). Due to the low viability of the flow cytometric assay after inactivation, the dead bacteria DNA was interfered after PMA treatment and could not be amplified. Therefore, we evaluated the structure of DO and the PMA-treated original donor microbiota (DP) by 16S rRNA sequencing analysis. As shown in **Figure 2C**, the Observed, Shannon and evenness (J) indices were slightly reduced after PMA-treated, although the differences were not statistically significant. PCoA ordination based on Bray-Curtis distances between OTU abundance profiles shows that fecal samples after PMA were distinctly separated from the DO group (**Figure 2B**). At the genus level, a slightly increased abundance of *Prevotella_7*, *CAG-352*, and *Prevotella_2* and a slightly decreased abundance of *Faecalibacterium* and *Veillonella* were observed after PMA-treated in comparison to the DO group (**Figure 2D**). Notably, at the family level, a distinct decrease in the abundance of Ruminococcaceae, Lachnospiraceae and Veillonellaceae and an increase in the abundance of Prevotellaceae and Bacteroidaceae were observed after eradication compared to before eradication or confirmation (**Figure S1**). Also, we collected 11 donor stool samples to explore the correlation between bacterial load and CT values using PMA-qPCR technique (Supplementary Material). As shown in **Figure S2**, the activity and total bacterial load were verified by fitting standard curves based on the CT values of qPCR and flow cytometry bacterial counts. The CT values gradually decreased as the total bacterial load increased, and the correlation coefficient between their total bacterial load and CT values was close to 1 (R^2^ = 0.90), indicating that the correlation between bacterial load and CT values was significant.

### The histological and viable gut community differences between DSS and the controls

**Figure 3:**
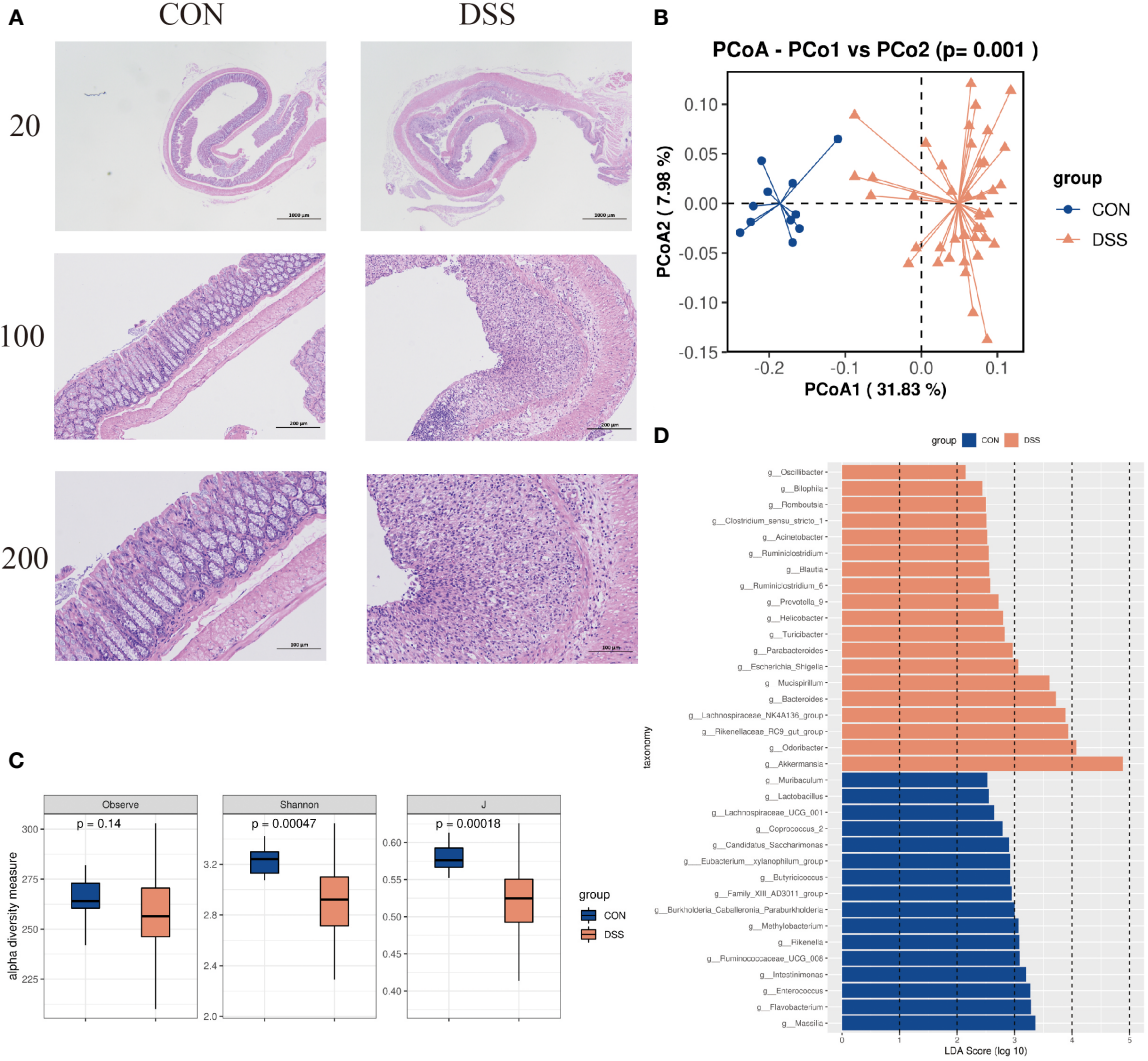
**(A)** HE dyeing experiment pictures. **(B)** Bacterial beta diversity. Principal Coordinates Analysis based on Bray-Curtis distances between the gut microbiota profiles of mice from the two groups. **(C)** Alpha diversity based on species richness, the Shannon diversity index, and the Inverse Simpson diversity index(J) in DSS and CON. **(D)** Significantly enriched bacterial taxa in the different groups as determined by LEfSe analysis (LDA sore >2).

Histological analysis showed that compared with normal mice, DSS-induced mice formed ulcerative foci in the mucosal layer of the colon, with necrosis spreading to the entire mucosa resulting in loss of lamina propria and proliferation of connective tissue, with varying numbers of inflammatory cells infiltrating between them and dilated crypt foci near the ulcerative foci (**Figure 3A**). In addition, 16S rRNA gene high-throughput sequencing analysis showed that the alpha diversity indices Observe, Shannon and J were significantly decreased in UC mice compared to CON group (*p*<0.05, **Figure 3C**). We observed clear the clustering of microbial communities between colitis mice and normal group by the PCoA plot (*p*<0.05, **Figure 3B**). Further, the microbiota composition in the DSS-induced colitis mice displayed a significantly different profile at genus level from that in the controls. Sixteen taxa including *Massilia*, *Rikenella*, *Butyricicoccus*, and *Enterococcus* were decreased in cases compared to CON group, while 19 genera including *Akkermansia*, *Blautia* and *Odoribacter* were significantly increased in DSS group (*p*<0.05, **Figure 3D**). These results indicate that the intestinal microbiota and colonic mucosal structure of mice were significantly changed by DSS induction.

### Effect of FMT on the composition of the gut microbiome

**Figure 4:**
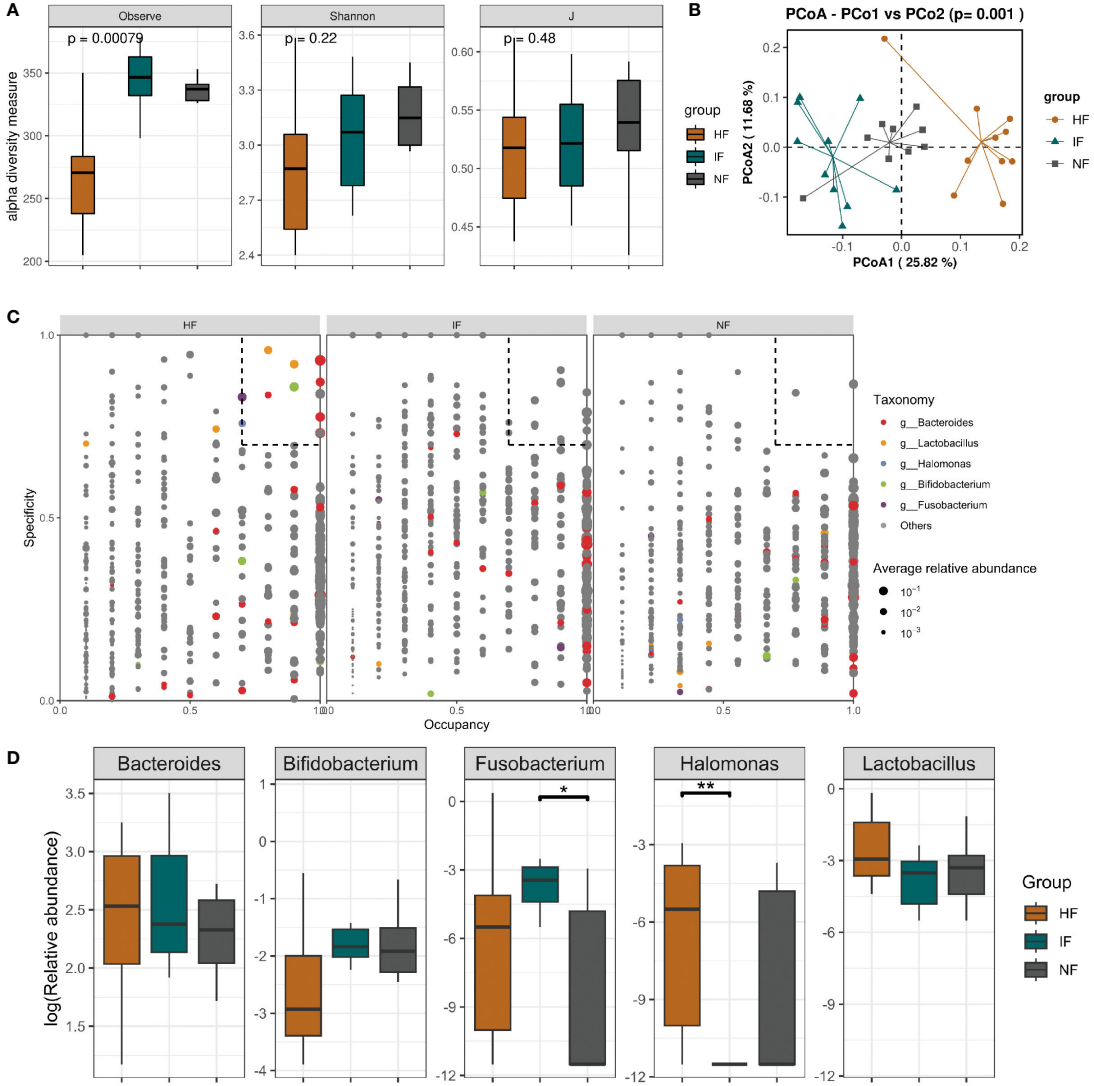
**(A)** Alpha diversity based on species richness, the Shannon diversity index, and J in HF, IF and NF. **(B)** Diagram of the Bray–Curtis distance principal coordinate analysis. **(C)** The SPEC-OCCU plots show the most abundant OTUs corresponding genera in HF; the x-axis represents occupancy; and the y-axis represents specificity. **(D)** The relative abundance of 5 specific genera among three groups. **p < 0.01 and *p < 0.05.

Acute colitis was induced in mice with 3.0% DSS and transplanted with DO, DI group and saline respectively. Subsequently, changes in the gut microbiota of the HF, NF and IF groups were analyzed by 16S rRNA gene high-throughput sequencing. Due to the failure of library construction for one sample, only 9 mice in NF group were included in the microbiota analysis. The Observed index of the HF group was significantly lower than that of the IF and NF group, but the Shannon and evenness indices were not significantly different (**Figure 4A**). We also found 70 genera specific to the IF group such as *Coprobacter*, *Eggerthella* and *Erysipelatoclostridium*, which may have contributed to the elevated IF group diversity (**Table S1**). Pre- and post-transplantation analysis of the three groups showed no obvious change in the observed index in the HF group compared with DSSHF group, while the index was markedly change in either IF vs DSSIF or NF vs DSSNF group (**Figures S1A**–**3A**). PCoA plots analysis showed distinct differences in the gut microbiota of the three groups after treatment (*p*<0.05, **Figure 4B**). As shown in **Figures S3B**–**5B**, the gut microbiota community structure of DSS-induced mice was changed after FMT (HF, IF and NF). In addition, SPEC-OCCU plots were analyzed for microbiota in the HF, IF and NF groups (**Figure 4C**). Five specific genera, *Bacteroides*, *Lactobacillus*, *Halomonas*, *Bifidobacterium* and *Fusobacterium*, were identified by analyzing specificity and occupancy (≥0.7) in the HF group compared to the NF and IF groups. The relative abundance of *Fusobacterium* was significantly higher in the IF group than in the NF group (*p*<0.05), and the HF group was not significantly different from the other two groups. However, the relative abundance of *Halomonas* and *Lactobacillus* were significantly higher in the HF group than in the IF group (**Figure 4D**). The distinct differences in taxa were observed after FMT compared to before FMT. A similar trend of *Bacteroides*, *Lactobacillus*, *Halomonas*, *Bifidobacterium* and *Fusobacterium* abundance was also observed in the before and after FMT group (**Figures S3C**-**5C**).

## Discussion

In this study, we conducted a FMT trial to evaluate the importance and shifts of viable gut microbiota in DSS-induced UC mice treated by FMT from a donor sample. A total of 30 mice were divided equally into 3 groups according to the different treatments, and another 10 mice without DSS inducement were set as control. By 16S rRNA gene sequencing analysis of fecal samples treated by PMA, we found that the structure of viable bacteria DNA is different from total bacteria DNA. The intestinal viable bacteria and colonic mucosal structure of mice were significantly changed by DSS induction. The histological analysis showed that FMT with live microbiota (HF) were able to improve colonic inflammatory conditions and colonic damage, whereas effect of dead microbiota was similar with the placebo with saline. Meanwhile, we identified key genera that changed after transplantation with HF, including *Bacteroides*, *Lactobacillus*, *Halomonas*, *Bifidobacterium* and *Fusobacterium*, which provides a reference for the treatment of UC.

We found that the bacterial structure of total DNA in donor fecal sample differed from the microbial community structure after PMA. As known, most of the microorganisms in the intestinal tract are difficult to be cultured by conventional methods (Costello et al., 2015). For general molecular methods, total DNA of a sample was used as a template for PCR amplification, which is difficult to distinguish viable and dead microorganisms, resulting in false negative results. With the development of powerful and convenient high-throughput sequencing technology, 16s rRNA gene or metagenomic sequencing is a common tool for measuring the relative abundance of specific microorganisms in microbial ecology (Reuter et al., 2015; Zemb et al., 2020). Therefore,the activity and profile of gut microbiota in donor samples can be thought as an important evaluation indicator in donor screening in the future.

The changes of viable microbiota were explored in the intestine of DSS-induced and normal mice. After DSS induction, HE staining showed that the intestinal tissues were damaged, accompanied by structural changes in the intestinal flora. Alpha diversity analysis and PCoA plots indicated that the composition of the intestinal live microbiota in colitis mice changed along with the altered intestinal tissue structure, and DSS induction disrupts the stable microenvironment of the intestine. Some live bacteria may play a central role. LEfSe confirmed our hypothesis by finding a total of 19 bacterial genera with large differences in DSS group, including *Akkermansia*, *Blautia* and *Odoribacter* et al. *Akkermansia* is known as a mucin-degrading bacterium with regulatory and inflammatory properties. DSS induced disruption of the mucosal layer in the hindgut and increased infiltration of acute inflammatory immune cells (Rinaldi et al., 2019). Meanwhile, the hypothesis of *Akkermansia* as an opportunistic bacterium that may flourish after ecosystem disruption (Machiels et al., 2020), which explained the increase of live *Akkermansia.* in the intestine of mice after DSS induction in normal mice. *Odoribacter* were also increased in DSS group. Li et al. found that *Odoribacter* showed a state of inhibition by other bacteria in healthy subjects, but were “unrestrained” and significantly more abundant in UC patients (Li et al., 2021b). The researchers also found a relationship between this opportunistic pathogen and pathophysiological mechanisms such as reduced SCFAs and increased inflammatory response. With a larger number of influential live bacteria identified through LEfSe method that may have potential significance for the diagnosis and treatment of UC and deserve to be further explored.

The result of DAI scores and histological examination indicated that live bacteria can participate in maintaining intestinal homeostasis, whereas dead bacteria often fail to play a role. The Observe index was significantly lower in the HF group than that in the IF and NF groups. This may be due to some dead bacteria from donor faeces, which interfered with the analysis of the live microbiota and caused differences in Observed index. 70 specific-genera was belong to the IF group such as *Coprobacter*, *Eggerthella* and *Erysipelatoclostridium*, which may have contributed to the elevated IF group diversity. The genus *Erysipelatoclostridium* is a pro-inflammatory microorganism with high potential to induce TH1 cells and high potential for intestinal inflammation (Nagayama et al., 2020). Bo Yang et al. found that *Eggerthella* may be associated with clinical symptoms of diarrhoea in a study on diarrhoeal irritable bowel syndrome and functional diarrhoea (Yang et al., 2021). Chen et al. found elevated relative abundance of *Escherichia-Shigella* in a study of the intestinal microbiota during acute necrotizing pancreatitis in rats (Chen et al., 2017). Furthermore, the PCoA plots showed that there were significant differences in the living microbiota of mice after different treatments. The differences of the intestinal structure tells that both live and dead bacteria were able to alter the intestinal structure of mice compared to the saline group. Combined with the HE staining results, the live bacteria was able to restore the intestinal health of mice, while the dead bacteria could not, probably because the dead bacteria could act as postbiotics to allow the growth of harmful bacteria.

Specificity and occupancy (≥0.7) were identified by SPEC-OCCU plots analysis of five specific genera in the HF group compared to the NF and IF groups, including *Bacteroides*, *Lactobacillus*, *Halomonas*, *Bifidobacterium* and *Fusobacterium*. In line with the He et al. study (He et al., 2013), *Bacteroides* was also substantially elevated in this trial. *Bacteroides* has good function on the improvement of endotoxaemia, reducing gut microbial lipopolysaccharide production and effectively inhibit pro-inflammatory immune responses, and low anthropoid bacteria can lead to inflammatory bowel disease (Althouse et al., 2019). Similarly, an increase in *Lactobacillus* was observed in UC mice after FMT treatment. Most of the current results prove that *Lactobacillus* is also the main genus used for the treatment of UC (Yun et al., 2020). For example, Liu et al. reduced intestinal lining inflammation by rectal enemas of *Lactobacillus*. It is inferred that *Lactobacillus* inhibits the onset of colitis in mice and may reduce the onset of stress-induced colitis (Liu et al., 2018). Liu et al. reported that *Halomonas* is the predominant genus associated with the jejunal and ileal mucosa of goats and speculated, and *Halomonas* may play a role in promoting immune development in the gut (Liu et al., 2019). Consistent with the present experiment, there was a substantial increase in live bacteria of the genus *Halomonas.* For *Bifidobacterium*, it has been used extensively in the treatment of inflammatory bowel diseases, such as UC (Xie et al., 2022). *Bifidobacterium* in human intestine can synthesise many vitamins such as vitamin B1/B2/B6, nikonic acid, pantothenic acid, folic acid and biotin. Once synthesised, these vitamins are then absorbed by the mucosal cells and contribute to the body’s metabolism and health maintenance (Huang et al., 2019). For *Fusobacterium*, it is a recognized pro-inflammatory bacterium that does not act in a simple one-way relationship with other bacteria, but may form mutually beneficial relationships that promote dysbiosis (microbial imbalance) in the community (Agarwal et al., 2020). From this we can infer that the live intestinal microbiota increased in beneficial bacteria, thus reducing the pro-inflammatory effect of *Fusobacterium*. In terms of relative abundance, the difference in *Bacteroides* and *Lactobacillus* between the groups was not significant, probably because the acute UC model was used for this modeling and the mice recovered naturally. That means live bacteria accelerate the healing of intestinal losses. So, higher levels of *Bacteroides*, *Lactobacillus*, *Halomonas* and *Bifidobacterium* in the live intestinal microbiota may be associated with the recovery of UC intestinal tissues, with the surviving live flora playing a major role. In addition to the vital importance of viable microbiota for the treatment of UC mice with FMT, SCFA produced by these microbiota may also play a key role in inhibiting intestinal inflammation, anti-tumor effects and regulating immune response (Mirsepasi-Lauridsen, 2022). For example, *Bifidobacterium* was known to produce the acetate that can protect against enteric infection in mice (Rabbani et al., 1999; Fukuda et al., 2011; Sepúlveda et al., 2018); *Bacteroides* was also the main bacteria involved in producing SCFA and play an important role (Kaakoush et al., 2014; Comstock, 2009). *Lactobacillus* produced butyrate by altering the intestinal microbiota, which maintains homeostasis in the gut, reduces inflammatory responses and serves as a source of energy for the renewal of intestinal epithelial cells (Jhun et al., 2021; Tian et al., 2019; Jena et al., 2020). As shown in **Figure S6**, it was found that SCFA-producing genera in the donor, such as *Bifidobacterium* and *Bacteroides* that colonized mice, may play a key role in the treatment of UC mice.

However, the current dose of FMT is based on the weight of the bacterial sludge (Zhang et al., 2020). The number of live organisms in the slurry is a key factor in judging the merit of the FMT product as well as its effectiveness in improving efficacy while reducing the number of doses taken by the patient and making it less difficult for the patient to take the medicine. Therefore, high bacterial level is the key to the efficacy of FMT. The usual analysis of total bacteria indicates is inaccuracy and can result in false positive results, so the live microbiota analysis its biological significance is greater compared to the total microbiota analysis. What’s more, many UC-associated inflammatory factors have been reported and it is important to explore the mechanisms of inflammation by detecting and observing changes in these inflammatory factors (Rubin et al., 2019). As most of the signaling pathways are significantly affected by the disease, further exploration to detect the expression of key factors in the signaling pathways can follow. The interrelationship between inflammatory factors and signaling pathways merits further investigation, which may provide further insights into targeting the microbial groups as a therapeutic strategy for UC and other diseases associated with the gut microbiota.

In summary, by H&E stained and 16S rRNA gene sequencing analysis of 30 DSS-induced mice and 10 controls with PMA treatment, we observed the significantly difference and identified UC-related viable genera in groups. After treatment of UC mice with DO, DI and raw saline group transplants, it was found that live bacteria played a key role in the treatment of UC mice. Most importantly, it would be useful to assess the composition of donor transplant material by viability assays to ensure that the microbiota composition includes a broad range of live bacteria, some of which may be important in mediating the therapeutic efficacy of FMT for microbiome-related disease. Therefore, we recommend that the activity of donor microbiota should be considered in FMT, and that detailed analysis of the types and numbers of live bacteria transplanted is essential to understanding the mechanisms of which FMT produces or fails to produce therapeutic effects.

## Data availability statement

The datasets presented in this study can be found in NCBI under accession number PRJNA911460.

## Ethics statement

The animal study was reviewed and approved by Animal Care and Ethics Committee of Fujian University of Traditional Chinese Medicine Laboratory Animal Center.

## Author contributions

YL, HL, MC, and JL conducted the mice experiments, managed the participants, interpreted the data, and drafted and reviewed the manuscript. MC, HT, and BZ analyzed the microbiota samples, analyzed the microbiome data, interpreted the data, and reviewed and contributed to the manuscript. HL, TL, and WX analyzed the tissue samples, analyzed the data, interpreted the data, and reviewed and contributed to the manuscript. AL, HW, MC, HL, and JH performed the analyses and sample processing, interpreted the data, and reviewed and contributed to the manuscript. YL, HT, and BZ conceived the study, analyzed the microbiome data, interpreted the data, and reviewed and contributed to the manuscript. All authors contributed to the article and approved the submitted version.
