# Supplementary material for: Shifts and importance of viable bacteria in treatment of DSS-induced ulcerative colitis mice with FMT
Source: Front Cell Infect Microbiol. 2023 Feb 6;13:1124256. doi: 10.3389/fcimb.2023.1124256 (PMC9939747; doi:10.3389/fcimb.2023.1124256)
Supplement: Supplementary file 1 [file DataSheet_1.docx]

Supplementary Material

Shifts and importance of viable bacteria in treatment of DSS-induced ulcerative colitis mice with FMT

**Jinglong Liu^1†^, Hao Lin^2†^, Man Cao^3,4†^, Tan Lin^2^, Aiqiang Lin^3^, Wei Xu^2,3^, Han Wang^3^, Jianquan He^3^, Yuantao Li^3*^, Hailing Tang^5*^, Bangzhou Zhang^3,4,6*^**

^1^Department of Gastroenterology, Shanxi Provincial People's Hospital, Shanxi, China

^2^Xiamen Institute of Union Respiratory Health, Xiamen, China

^3^Center for Research and Development, Xiamen Treatgut Biotechnology Co., Ltd., Xiamen, China

^4^Department of Gastroenterology, The Second Affiliated Hospital of Fujian University of Traditional Chinese Medicine, Fuzhou, China

^5^Division of Gastroenterology, Xi'an Central Hospital, Xi'an, China

^6^School of Pharmacy, Fujian University of Traditional Chinese Medicine, Fuzhou, China

^†^These authors have contributed equally to this work and share first authorship

*** Correspondence:**

Corresponding Author

liyuantao@treatgut.com (YT. Li), tanghl@yeah.net (HL. Tang), geebzbz@xmu.edu.cn (BZ. Zhang)

# Supplementary Data

In this study of absolute quantification of live bacteria using the PMA-qPCR technique, we explored the correlation between bacterial load and CT values. Twelve donor faecal samples were collected and the activity and bacterial load of the donor faeces were first measured by flow cytometry, and tenfold gradient dilutions were performed based on the bacterial content obtained by flow cytometry. Five gradient samples (10, 10^2^, 10^3^, 10^4^, 10^5^) were prepared by dilution, 1 ml per gradient, in sterile 1.5 ml centrifuge tubes for PMA treatment, and faecal samples were extracted after treatment DNA was then measured using qPCR to obtain CT values. A standard curve was obtained based on the CT values from qPCR and flow cytometry bacterial counts, which were validated for viability and total bacterial counts.

**Construction of a Standard Curve**

The fluorescent stains samples with a LIVE/DEAD™ BacLight™ Bacterial Viability Kit. The bacteria per milliliter were transformed using the functions of log represented on the abscissa. After qPCR, similar Ct values ordinate was established.

# Supplementary Figures and Tables

## Supplementary Figures


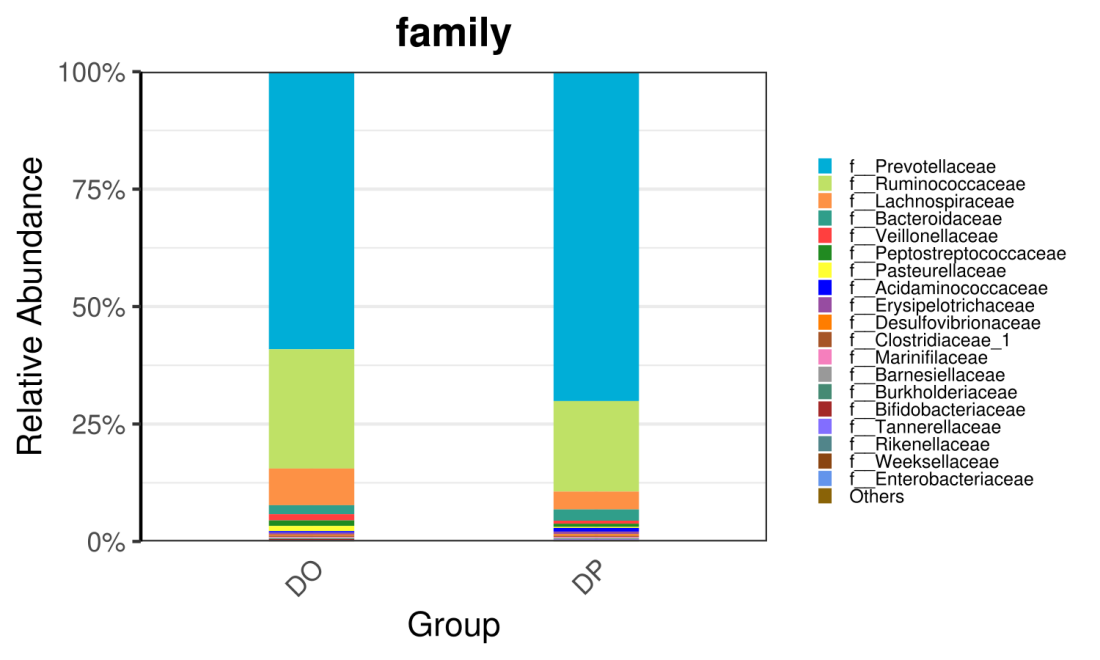


**Supplementary Figure 1.** The composition of the gut microbiota between DO and DP group at the family level (the abundance of Top 20 are shown).


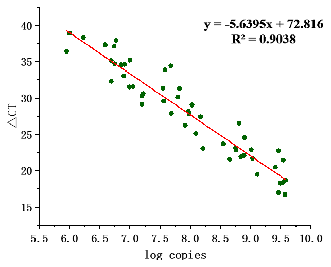


**Supplementary Figure 2.** Establishment of the fitted standard curve. The horizontal coordinate is the logarithm of the total number of bacteria per mL (cells/mL) and the vertical coordinate is the ct value after qPCR with sample DNA gradient dilution as template.


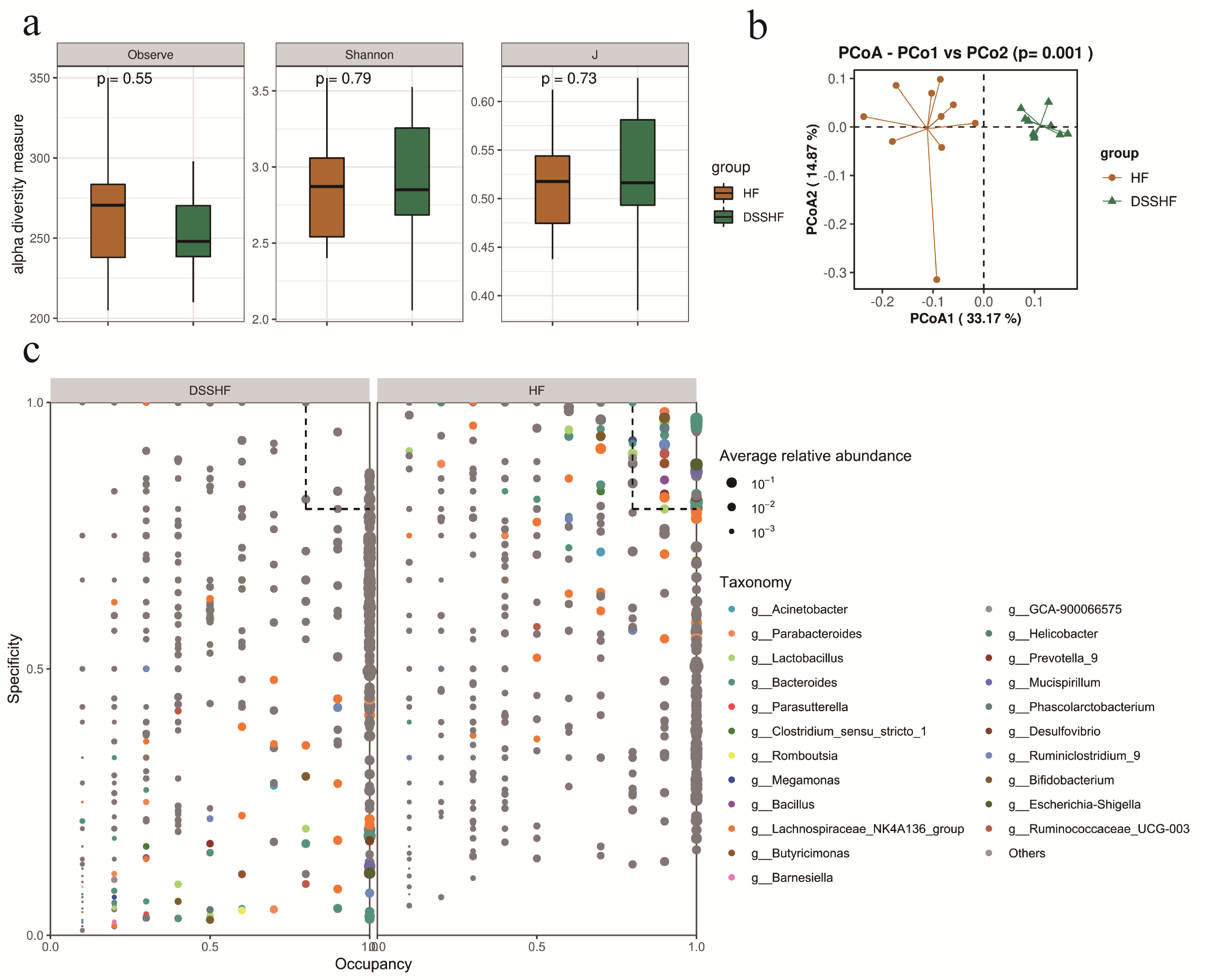


**Supplementary Figure 3.** (a) Alpha diversity based on species richness, the Shannon diversity index, and the Inverse Simpson diversity index(J) in DSSHF and HF.(b)Bacterial beta diversity. Principal Coordinates Analysis based on Bray-Curtis distances between the gut microbiota profiles of individuals from the two groups. (c) The SPEC-OCCU plots show the most abundant OTUs corresponding genera in HF; the x-axis represents occupancy; and the y-axis represents specificity.


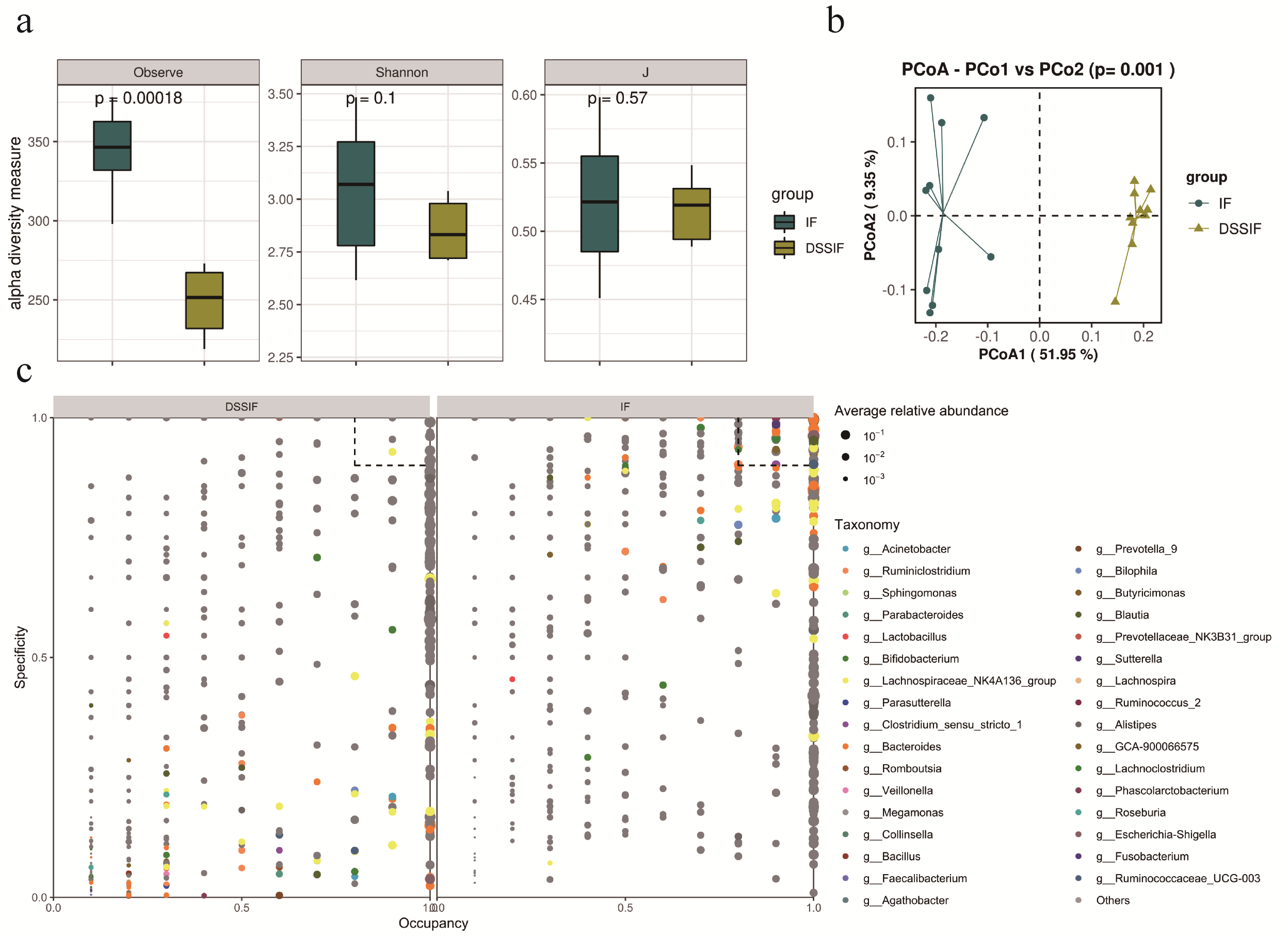


**Supplementary Figure 4.** (a) Alpha diversity based on species richness, the Shannon diversity index, and the Inverse Simpson diversity index(J) in DSSIF and IF.(b)Bacterial beta diversity. Principal Coordinates Analysis based on Bray-Curtis distances between the gut microbiota profiles of individuals from the two groups. (c) The SPEC-OCCU plots show the most abundant OTUs corresponding genera in IF; the x-axis represents occupancy; and the y-axis represents specificity.


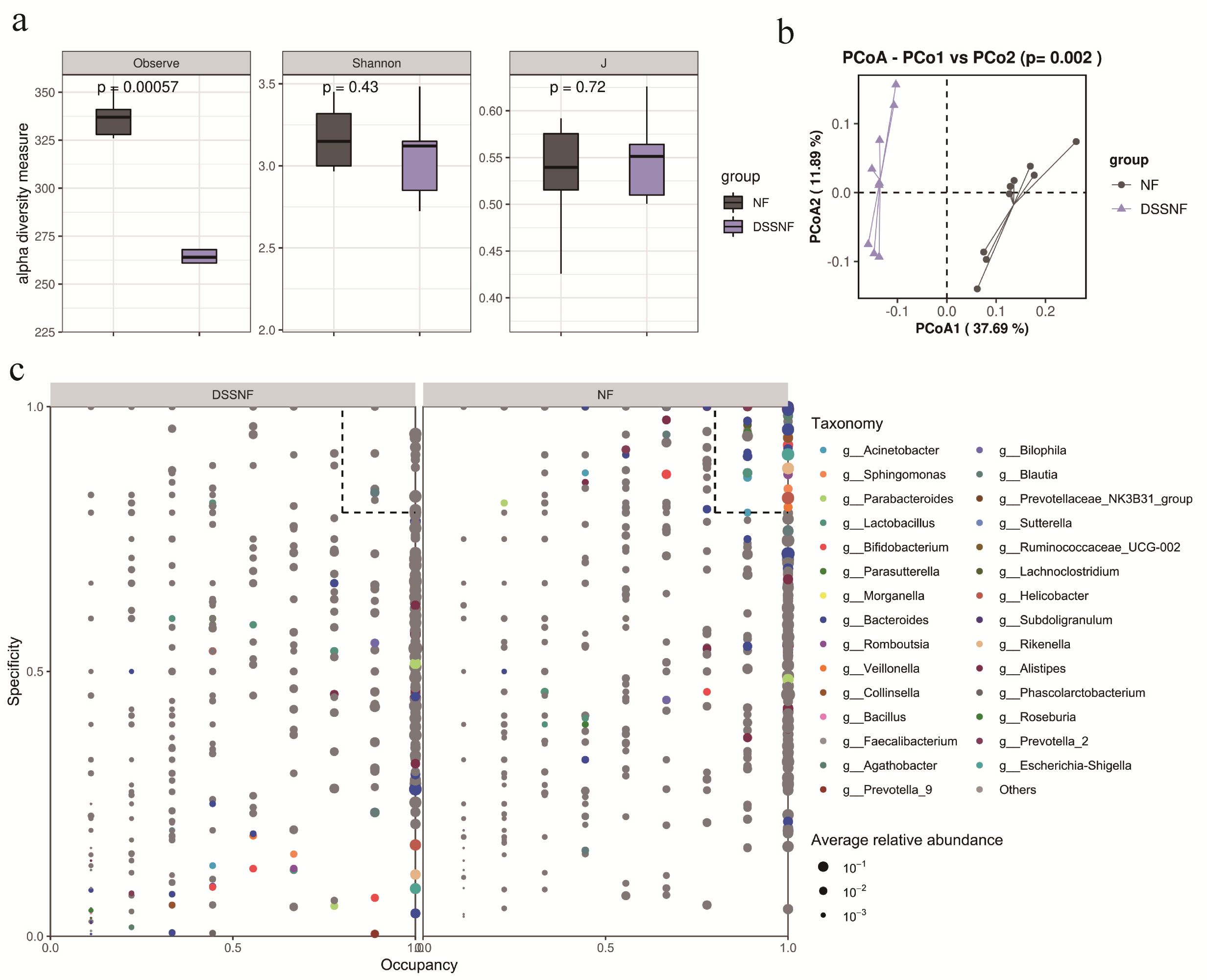


**Supplementary Figure 5.** (a) Alpha diversity based on species richness, the Shannon diversity index, and the Inverse Simpson diversity index(J) in DSSNF and NF.(b)Bacterial beta diversity. Principal Coordinates Analysis based on Bray-Curtis distances between the gut microbiota profiles of individuals from the two groups. (c) The SPEC-OCCU plots show the most abundant OTUs corresponding genera in NF; the x-axis represents occupancy; and the y-axis represents specificity.


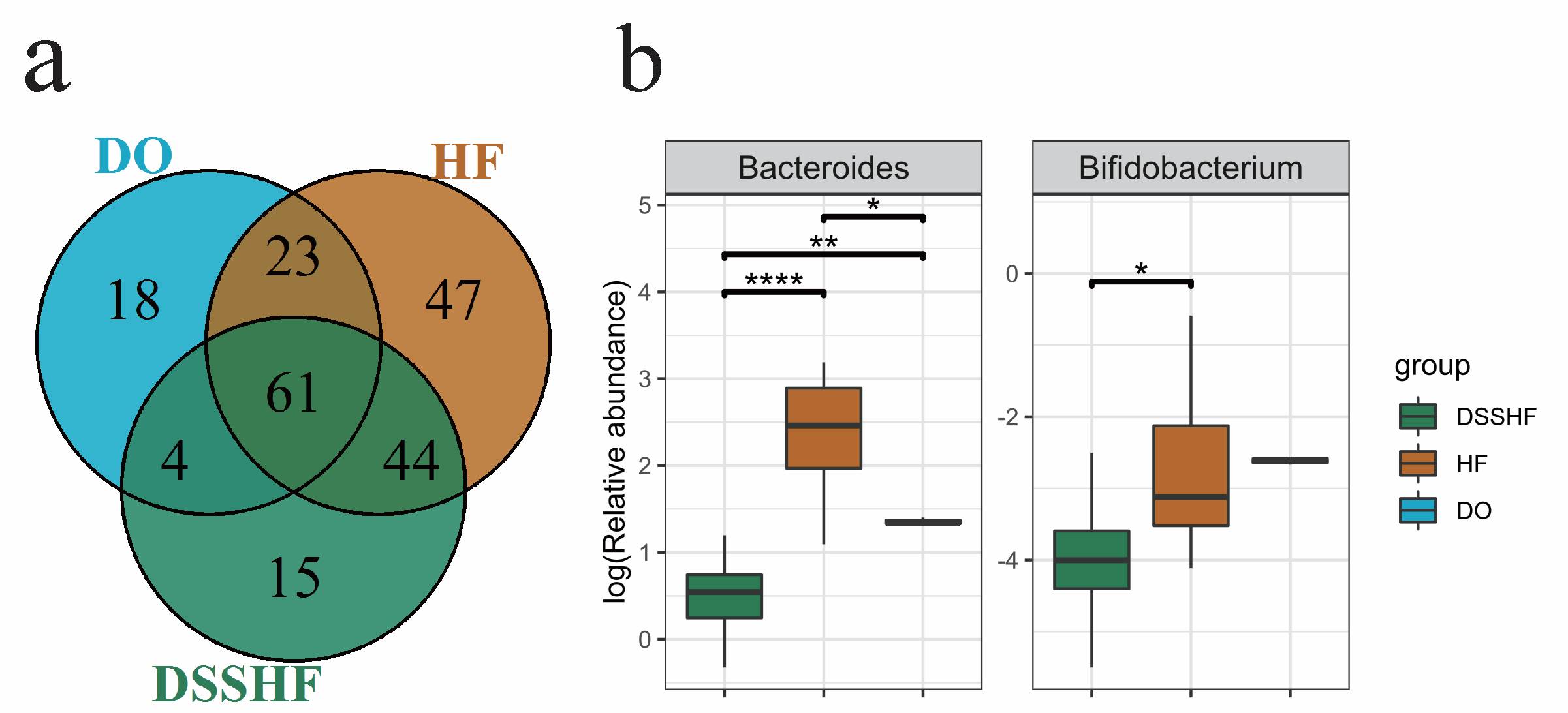


**Supplementary Figure 6.** (a) The Venn diagram illustrates all overlapping genera identified in the DO, DSSHF (samples from the mice that would receive the DO transplant (before transplant)) and HF groups. (b) The relative abundance of 2 specific genera among DO, DSSHF and HF groups.

## Supplementary Tables

Table 1. Compared to HF and NF groups, the IF group was able to annotate to the genus level with a specific display of the unique 70 OTUs.

| **OTUs** | **taxonomy** |
| --- | --- |
| OTU_1191 | f__Lachnospiraceae;g__[Eubacterium]_ventriosum_group;s__un_g_[Eubacterium]_ventriosum_group |
| OTU_1355 | f__Lachnospiraceae;g__[Ruminococcus]_torques_group;s__un_g_[Ruminococcus]_torques_group |
| OTU_593 | f__Lachnospiraceae;g__[Ruminococcus]_torques_group;s__un_g_[Ruminococcus]_torques_group |
| OTU_784 | f__Ruminococcaceae;g__[Eubacterium]_coprostanoligenes_group;s__un_g_[Eubacterium]_coprostanoligenes_group |
| OTU_501 | f__Acidaminococcaceae;g__Acidaminococcus;s__un_g_Acidaminococcus |
| OTU_654 | f__Spirosomaceae;g__Arcicella;s__un_g_Arcicella |
| OTU_1141 | f__Bacteroidaceae;g__Bacteroides;s__un_g_Bacteroides |
| OTU_1296 | f__Bacteroidaceae;g__Bacteroides;s__un_g_Bacteroides |
| OTU_1415 | f__Bacteroidaceae;g__Bacteroides;s__un_g_Bacteroides |
| OTU_568 | f__Bacteroidaceae;g__Bacteroides;s__un_g_Bacteroides |
| OTU_1456 | f__Lachnospiraceae;g__Blautia;s__un_g_Blautia |
| OTU_1619 | f__Lachnospiraceae;g__Blautia;s__un_g_Blautia |
| OTU_521 | f__Lachnospiraceae;g__Blautia;s__un_g_Blautia |
| OTU_1551 | f__Brevibacteriaceae;g__Brevibacterium;s__un_g_Brevibacterium |
| OTU_420 | f__Burkholderiaceae;g__Burkholderia-Caballeronia-Paraburkholderia;s__un_g_Burkholderia-Caballeronia-Paraburkholderia |
| OTU_630 | f__Ruminococcaceae;g__Butyricicoccus;s__un_g_Butyricicoccus |
| OTU_758 | f__Marinifilaceae;g__Butyricimonas;s__un_g_Butyricimonas |
| OTU_652 | f__Christensenellaceae;g__Christensenellaceae_R-7_group;s__un_g_Christensenellaceae_R-7_group |
| OTU_1304 | f__Barnesiellaceae;g__Coprobacter;s__un_g_Coprobacter |
| OTU_529 | f__Lachnospiraceae;g__Coprococcus_2;s__un_g_Coprococcus_2 |
| OTU_338 | f__Propionibacteriaceae;g__Cutibacterium;s__un_g_Cutibacterium |
| OTU_295 | f__Erysipelotrichaceae;g__Dubosiella;s__un_g_Dubosiella |
| OTU_749 | f__Eggerthellaceae;g__Eggerthella;s__un_g_Eggerthella |
| OTU_1151 | f__Rhizobiaceae;g__Ensifer;s__un_g_Ensifer |
| OTU_371 | f__Eggerthellaceae;g__Enterorhabdus;s__un_g_Enterorhabdus |
| OTU_874 | f__Eggerthellaceae;g__Enterorhabdus;s__un_g_Enterorhabdus |
| OTU_565 | f__Erysipelotrichaceae;g__Erysipelatoclostridium;s__un_g_Erysipelatoclostridium |
| OTU_1378 | f__Enterobacteriaceae;g__Escherichia-Shigella;s__un_g_Escherichia-Shigella |
| OTU_700 | f__Family_XIII;g__Family_XIII_AD3011_group;s__un_g_Family_XIII_AD3011_group |
| OTU_414 | f__Family_XIII;g__Family_XIII_UCG-001;s__un_g_Family_XIII_UCG-001 |
| OTU_1649 | f__Lachnospiraceae;g__Howardella;s__un_g_Howardella |
| OTU_581 | f__Melioribacteraceae;g__IheB3-7;s__un_g_IheB3-7 |
| OTU_354 | f__Lachnospiraceae;g__Lachnospiraceae_NK4A136_group;s__un_g_Lachnospiraceae_NK4A136_group |
| OTU_472 | f__Lachnospiraceae;g__Lachnospiraceae_NK4A136_group;s__un_g_Lachnospiraceae_NK4A136_group |
| OTU_738 | f__Lachnospiraceae;g__Lachnospiraceae_NK4A136_group;s__un_g_Lachnospiraceae_NK4A136_group |
| OTU_886 | f__Lachnospiraceae;g__Lachnospiraceae_NK4A136_group;s__un_g_Lachnospiraceae_NK4A136_group |
| OTU_364 | f__Lachnospiraceae;g__Lachnospiraceae_UCG-001;s__un_g_Lachnospiraceae_UCG-001 |
| OTU_1065 | f__Veillonellaceae;g__Megasphaera;s__un_g_Megasphaera |
| OTU_1221 | f__Thermaceae;g__Meiothermus;s__un_g_Meiothermus |
| OTU_466 | f__Beijerinckiaceae;g__Methylobacterium;s__un_g_Methylobacterium |
| OTU_832 | f__Beijerinckiaceae;g__Methylobacterium;s__un_g_Methylobacterium |
| OTU_1376 | f__Muribaculaceae;g__Muribaculum;s__un_g_Muribaculum |
| OTU_525 | f__Isosphaeraceae;g__Paludisphaera;s__un_g_Paludisphaera |
| OTU_739 | f__Tannerellaceae;g__Parabacteroides;s__un_g_Parabacteroides |
| OTU_682 | f__Moraxellaceae;g__Perlucidibaca;s__un_g_Perlucidibaca |
| OTU_1548 | f__Prevotellaceae;g__Prevotella_9;s__un_g_Prevotella_9 |
| OTU_940 | f__Rikenellaceae;g__Rikenellaceae_RC9_gut_group;s__un_g_Rikenellaceae_RC9_gut_group |
| OTU_491 | f__Lachnospiraceae;g__Roseburia;s__un_g_Roseburia |
| OTU_713 | f__Lachnospiraceae;g__Roseburia;s__un_g_Roseburia |
| OTU_829 | f__Lachnospiraceae;g__Roseburia;s__un_g_Roseburia |
| OTU_478 | f__Acetobacteraceae;g__Roseomonas;s__un_g_Roseomonas |
| OTU_626 | f__Micrococcaceae;g__Rothia;s__un_g_Rothia |
| OTU_764 | f__Ruminococcaceae;g__Ruminiclostridium_9;s__un_g_Ruminiclostridium_9 |
| OTU_1538 | f__Ruminococcaceae;g__Ruminococcaceae_NK4A214_group;s__un_g_Ruminococcaceae_NK4A214_group |
| OTU_976 | f__Ruminococcaceae;g__Ruminococcaceae_NK4A214_group;s__un_g_Ruminococcaceae_NK4A214_group |
| OTU_667 | f__Ruminococcaceae;g__Ruminococcaceae_UCG-002;s__un_g_Ruminococcaceae_UCG-002 |
| OTU_963 | f__Ruminococcaceae;g__Ruminococcaceae_UCG-002;s__un_g_Ruminococcaceae_UCG-002 |
| OTU_1712 | f__Ruminococcaceae;g__Ruminococcaceae_UCG-009;s__un_g_Ruminococcaceae_UCG-009 |
| OTU_572 | f__Ruminococcaceae;g__Ruminococcaceae_UCG-010;s__un_g_Ruminococcaceae_UCG-010 |
| OTU_229 | f__Ruminococcaceae;g__Ruminococcus_1;s__un_g_Ruminococcus_1 |
| OTU_1449 | f__Eggerthellaceae;g__Slackia;s__un_g_Slackia |
| OTU_645 | f__Spirosomaceae;g__Spirosoma;s__un_g_Spirosoma |
| OTU_373 | f__Family_XVII;g__Thermaerobacter;s__un_g_Thermaerobacter |
| OTU_1145 | f__Xanthomonadaceae;g__Thermomonas;s__un_g_Thermomonas |
| OTU_528 | f__Thermaceae;g__Thermus;s__un_g_Thermus |
| OTU_253 | f__Chitinophagaceae;g__Vibrionimonas;s__un_g_Vibrionimonas |
| OTU_1111 | f__Lachnospiraceae;g__Lachnospiraceae_NK4A136_group;s__un_g_Lachnospiraceae_NK4A136_group |
| OTU_1190 | f__Peptostreptococcaceae;g__Peptostreptococcus;s__un_g_Peptostreptococcus |
| OTU_1014 | f__Lachnospiraceae;g__Tyzzerella_3;s__un_g_Tyzzerella_3 |
